# Phase Ib Trial of Copanlisib, A Phosphoinositide-3 Kinase (PI3K) Inhibitor, with Trastuzumab in Advanced Pre-Treated HER2-Positive Breast Cancer “PantHER”

**DOI:** 10.3390/cancers13061225

**Published:** 2021-03-11

**Authors:** Niamh M. Keegan, Simon J. Furney, Janice M. Walshe, Giuseppe Gullo, M. John Kennedy, Diarmuid Smith, John McCaffrey, Catherine M. Kelly, Keith Egan, Jennifer Kerr, Mark Given, Peter O’Donovan, Andres Hernando, Ausra Teiserskiene, Imelda Parker, Elaine Kay, Angela Farrelly, Aoife Carr, Giulio Calzaferri, Ray McDermott, Maccon M. Keane, Liam Grogan, Oscar Breathnach, Patrick G. Morris, Sinead Toomey, Bryan T. Hennessy

**Affiliations:** 1 Department of Molecular Medicine, Royal College of Surgeons in Ireland, Dublin, Ireland; keegannm@tcd.ie (N.M.K.); angelafarrelly@rcsi.ie (A.F.); aoifecarr@rcsi.ie (A.C.); Bryan.Hennessy@hse.ie (B.T.H.); 2 Department of Medical Oncology, Beaumont Hospital, Dublin, Ireland; liamgrogan@beaumont.ie (L.G.); osbreathnach@beaumont.ie (O.B.); patrickmorris@beaumont.ie (P.G.M.); 3 Cancer Clinical Trials & Research Unit, Beaumont Hospital, Dublin, Ireland; keithegan2@beaumont.ie; 4 Genomic Oncology Research Group, Department of Physiology & Medical Physics, Centre for Systems Medicine, Royal College of Surgeons in Ireland, Dublin, Ireland; simonfurney@rcsi.ie (S.J.F.); didonov@tcd.ie (P.O.); 5 Department of Medical Oncology, St Vincent’s University Hospital, Dublin, Ireland; Janice.walshe@amnch.ie (J.M.W.); G.Gullo@st-vincents.ie (G.G.); Giulio.Calzaferri@ccrt.ie (G.C.); RAY.MCDERMOTT@tuh.ie (R.M.); 6 Cancer Trials Ireland, Innovation House, Glasnevin, Dublin, Ireland; jkennedy@stjames.ie (M.J.K.); jmccaffrey@materprivate.ie (J.M.); ckelly@mater.ie (C.M.K.); Andres.Hernando@cancertrials.ie (A.H.); Ausra.Teiserskiene@cancertrials.ie (A.T.); Imelda.Parker@cancertrials.ie (I.P.); Maccon.Keane@hse.ie (M.M.K.); 7 Department of Medical Oncology, St James’s Hospital, Dublin, Ireland; 8 Department of Endocrinology, Beaumont Hospital, Dublin, Ireland; diarmuidsmith@beaumont.ie; 9 Department of Medical Oncology, Mater Misericordia University Hospital, Dublin, Ireland; 10 Department of Radiology, Beaumont Hospital, Dublin, Ireland; jenniferkerr@beaumont.ie (J.K.); markgiven@beaumont.ie (M.G.); 11 Department of Pathology, Beaumont Hospital, Dublin, Ireland; elainekay@beaumont.ie; 12 Department of Medical Oncology, Galway University Hospital, Galway, Ireland

**Keywords:** phosphoinositide-3 kinase (PI3K), trastuzumab, breast neoplasms, maximum tolerated dose, PIK3CA protein, human, circulating tumour DNA

## Abstract

**Simple Summary:**

Patients with HER-2 positive breast cancer who progress through available HER2-targeted therapy, at present, have few effective treatment options. PIK3CA is mutated in approximately 20% of HER2 positive breast cancers, contributes to HER-2 therapy resistance and may be predictive of response to PI3K inhibitors, including copanlisib. PIK3CA gene mutations were assessed in archival tumour tissue and serially in plasma circulating tumour DNA over the course of treatment with copanlisib. Disease stabilisation (stable disease ≥16 weeks) was seen with copanlisib and trastuzumab in a proportion of participants (n = 6, 50%). PIK3CA mutation detected in archival tumour tissue did not appear to predict tumour response to copanlisib and trastuzumab in this small, heavily pre-treated cohort. Notably, PIK3CA circulating tumour DNA mutations were detected in the plasma of all trial participants, including those who tested negative for the mutation in tissue. This study established a dosing strategy for the novel combination of the PI3K inhibitor copanlisib with trastuzumab and suggested clinical activity for the combination in heavily pre-treated HER-2 positive advanced breast cancer. Further evaluation in a phase 2 study in patients with HER2 therapy resistant tumours is ongoing (NCT02705859).

**Abstract:**

Background: Activation of the phosphoinositide-3 kinase (PI3K) pathway is a resistance mechanism to anti-human epidermal growth factor receptor 2 (HER2) therapy. This phase Ib trial was conducted to determine the maximum tolerated dose (MTD) of copanlisib, an intravenous (IV) pan-class I PI3K inhibitor, combined with trastuzumab. Methods: Patients with advanced HER2-positive breast cancer and disease progression following at least one prior line of HER2 therapy in the metastatic setting were treated with copanlisib (45 or 60 mg) IV on days 1, 8 and 15 of a 28-day cycle with a fixed dose of trastuzumab 2 mg/kg weekly. Results: Twelve patients were enrolled. The MTD was determined as copanlisib 60 mg plus trastuzumab 2 mg/kg weekly. The most common adverse events of any grade occurring in more than two patients were hyperglycaemia (58%), fatigue (58%), nausea (58%) and hypertension (50%). Stable disease was confirmed at 16 weeks in six participants (50%). *PIK3CA* mutations were detected in archival tumour of six participants (50%). *PIK3CA* hotspot mutations, were detectable in pre- and on-treatment plasma of all participants. Pre- and post-treatment tumour biopsies for two patients identified temporal genomic heterogeneity, somatic mutations in the *TRRAP* gene, which encodes a PI3K-like protein kinase, and emergent somatic mutations related to protein kinase signalling. Conclusion: Copanlisib and trastuzumab can be safely administered with fair overall tolerability. Preliminary evidence of tumour stability was observed in patients with heavily pre-treated, metastatic HER2 positive breast cancer. Several potential biomarkers were identified for further study in the current phase 2 clinical trial. NCT: 02705859.

## 1. Introduction

The human epidermal growth factor receptor 2 (HER2) gene is amplified or overexpressed in approximately 20% of breast cancers and was previously associated with a poorer prognosis. The advent of a range of HER2 targeted therapies, including the monoclonal antibody trastuzumab, significantly improved outcomes for patients with this subtype of breast cancer but only about one third of women demonstrate tumour regression with trastuzumab monotherapy and many patients treated with trastuzumab plus chemotherapy still develop progressive disease within one year [1,2,3]. For those with HER-2 positive breast cancer and resistance to trastuzumab-based therapy, there are relatively few effective treatment options. Outcomes have been improved by the addition or sequencing of further anti HER2 therapies, supporting the idea that continued suppression of HER2 is important following trastuzumab resistance [4,5]. Additionally, molecular studies have proven persistent expression of HER-2 alongside emergence of markers associated with HER2 therapy resistance [6]. 

Multiple mechanisms for trastuzumab therapy resistance have been proposed, but because the anti-tumour activity of HER2 inhibition at least partly depends on inhibition of the downstream phosphatidylinositol 3-kinase (PI3K) pathway, aberrant activation of this pathway can contribute to trastuzumab resistance [7,8]. Activation of PI3K leads to a downstream signalling cascade that promotes tumour cell survival, growth, metabolism, motility, and progression [9]. In at least one-quarter to one-third of HER2-positive breast cancers there are dysregulated, HER2-independent activation of the PI3K pathway by mutation of *PIK3CA* or loss of PTEN [10,11]. Frequent or hotspot mutations of *PIK3CA*, which encodes the catalytic subunit of PI3K, are found at exon 9 (E542K and E545K) and exon 20 (H1047R) [12].

PIK3CA mutation may be predictive of response to PI3K inhibitor therapy [13,14,15]. Copanlisib (BAY 80-6946; Bayer Pharma AG, Berlin, Germany) is a highly selective, intravenous, pan-class I PI3K inhibitor that shows particular activity against PI3Kα, the isoform encoded by the *PIK3CA* gene, and against the PI3Kδ isoform [16]. In preclinical studies, the combination of copanlisib with trastuzumab was shown to be synergistic in HER2 therapy resistant cell lines [17]. We therefore undertook a phase Ib study to evaluate the safety and preliminary efficacy of copanlisib with trastuzumab for patients with trastuzumab resistant disease. Phase III trials have suggested that *PIK3CA* mutations may be predictive of response to PI3K inhibitor therapy in oestrogen receptor positive breast cancer [14]. In this trial, prospectively collected serial tumour and blood samples were analysed for *PIK3CA* and other mutations. 

## 2. Materials and Methods

### 2.1. Study Objectives

The primary objective was to determine the maximum tolerated dose (MTD) of copanlisib in combination with trastuzumab in an open label, single arm, adaptive multi-centre clinical trial. 

Secondary objectives included the assessment of the safety and tolerability of this combination. A preliminary evaluation of anti-tumour efficacy was also performed per Response Evaluation Criteria in Solid Tumours version 1.1 (RECIST v1.1) [18]. Exploratory objectives included the evaluation of *PIK3CA* mutations in archival tumour tissue and in circulating tumour DNA (ctDNA) derived from serial plasma samples collected over the duration of treatment. Next generation sequencing (NGS) was performed on available serial tumour biopsies. 

### 2.2. Eligibility

Eligible patients included women who were ≥18 years, with metastatic or incurable locally recurrent HER2 positive breast cancer as determined by immunohistochemistry (IHC) 3+ or fluorescence in situ hybridisation (FISH) or chromogenic in situ hybridisation (CISH) positive on at least one tumour sample (diagnostic breast biopsy, surgical breast resection sample or metastatic disease site biopsy) along with measurable disease per RECIST 1.1. There must have been tumour progression on at least one prior line of trastuzumab or Trastuzumab Emtansine (T-DM1)-based treatment in the advanced disease setting and any number of prior therapies was acceptable prior to this. Adequate organ function including fasting glucose ≤120 mg/dL (≤ 6.0 mmol/L) if not diabetic or < 160 mg/dL (≤ 8.9 mmol/L) if diabetic and an Eastern Cooperative Oncology Group (ECOG) performance status ≤ 2 were requirements. Patients with treated, controlled brain metastases were eligible.

Exclusion criteria included the presence of uncontrolled brain metastasis or seizure disorder, uncontrolled arterial hypertension or uncontrolled diabetes mellitus (HbA1c > 8.5%), active cardia disease or thromboembolic even, a non-healing wound, ulcer, or bone fracture, active clinically serious infections > Grade 2 or known HIV, Hepatitis B, C or Cytomegalovirus (CMV) positivity or uncontrolled intercurrent illnesses. Excluded concomitant therapies included radiotherapy or immuno-/chemotherapy fewer than 4 weeks before the start of study therapy, CYP3A4 inhibitors or inducers within 14 days prior to the start of study therapy, myeloid growth factors, blood or platelet transfusion less than 7 days before the start of study therapy or ongoing immunosuppressive treatment, steroid therapy at a daily dose higher than 16 mg prednisone or equivalent, or anti-arrythmics. Full details of inclusion and exclusion criteria are shown in Table S1. 

The study was approved by the Health Products Regulatory Authority of Ireland (HPRA) and a national ethics committee (Reference number ECM 3 (iii)) and followed the Declaration of Helsinki and Good Clinical Practice (GCP) guidelines. Written informed consent was required for enrolment.

### 2.3. Study Design and Treatment

#### Dosing and Administration

Eligible patients were assigned at registration to one of two dose levels: copanlisib 45 mg (dose level 1) or 60 mg (dose level 2) flat dosing IV on days 1, 8 and 15 of a 28-day cycle plus trastuzumab (4 mg/kg IV Cycle 1 Day 1 and then 2 mg/kg IV weekly starting from day 8). A starting dose of 45 mg copanlisib was determined based on data from single agent studies [19]. Patients were accrued in cohorts of 6 (6 + 6 design), with dose escalation and determination of MTD based on the occurrence of dose limiting toxicity (DLT) within the first 28 days using the usual threshold probability of 33%. DLTs were defined as the following: if deemed any treatment-emergent grade 3 or 4 non-hematologic toxicity other than nausea, vomiting or fatigue, any grade 4 thrombocytopenia (platelet count < 25 × 10^9^/L), grade 3 thrombocytopenia (platelet count 25 × 10^9^/L–< 50 × 10^9^/L) associated with bleeding, grade 4 neutropenia (absolute neutrophil count (ANC) < 0.5 × 10^9^/L) lasting > 4 days, febrile neutropenia defined as ANC < 1.0 × 10^9^/L concurrent with fever (with a single temperature of >38.3 °C (101°F) or a sustained temperature of >38 °C (100.4°F) for more than one hour, or inability to resume dosing for cycle 2 at the assigned dose level within 21 days due to treatment-related toxicity occurring during the first cycle (within 28 days from first dose) of treatment and possibly, probably or definitely related to the combination of copanlisib plus trastuzumab. The MTD was defined as the highest dose tested in which fewer than one third of patients, treated at that dose level, experienced a DLT(s) Supplement 1). A de-escalation copanlisib dose of 30 mg (dose level-1) was allowed if 45 mg was not tolerated. 

### 2.4. Study Assessments

#### 2.4.1. Safety

Safety assessments included glucose and blood pressure monitoring on the day of copanlisib infusion. The National Cancer Institute Common Terminology Criteria for Adverse Events (CTCAE version 4.0) (National Cancer Institute, Bethesda, MD, USA) was used to record severity and attribution of toxicities. Physical examination, vital signs, complete blood cell count, serum chemistry, and fasting glucose, were performed on days 1, 8, 15 and 22 of each cycle. There was a 12-lead electrocardiogram and cardiac evaluation with echocardiogram performed every 8 weeks. 

Pre-copanlisib glucose levels had to be ≤8.9 mmol/L (fasting) or ≤11.1 mmol/L (non-fasting) to proceed with treatment if participant had developed post infusion hyperglycaemia (glucose intolerance) of >13.9 mmol/L. A dose reduction in copanlisib by one dose level was mandatory in the event of one episode of asymptomatic hyperglycaemia >27.8 mmol/L (grade 4). Treatment was to be permanently discontinued in the case of recurrence of asymptomatic hyperglycaemia >27.8 mmol/L. Post infusion hyperglycaemia required participants to have a repeat capillary glucose within 24 h and to continue monitoring fasting glucose until resolution of hyperglycaemia. 

Patients with pre-existing arterial hypertension were required to adhere to their regular medication schedule and take their usual doses on the days of study drug infusion. If pre copanlisib blood pressure exceeded 150/90, there was no dose given. In the event of the occurrence of arterial hypertension ≥160/100 mmHg during infusion of copanlisib at any cycle, antihypertensive treatment prompted an interruption of the infusion and administration of an antihypertensive that did not interact with CYP3A4. The management of acute arterial hypertension following copanlisib was individualised for each patient, and although no antihypertensive treatment was specified in the study protocol, experience from Phase I studies suggested the benefit of dihydropyridine calcium channel blockers, including amlodipine and felodipine. Topical nitrates were also recommended. Verapamil and diltiazem (non-dihydropyridine calcium channel blockers) were not allowed to be administered due to a potential CYP3A4 interaction. In the event of the occurrence of Grade 3 arterial hypertension (≥160/100 mmHg) during infusion of copanlisib, the infusion was interrupted, and antihypertensive treatment as suggested above was administered. The infusion was resumed when blood pressure returned to <150/90 mmHg. 

Dermatologic toxicity was managed with topical emollients, steroid or topical antibiotics with the addition of antihistamine for grade 2 or greater pruritis. Grade 2 rash led to dose interruption until reduced to ≤grade 1. On the 2nd appearance of grade 2 rash or on first appearance of higher-grade rash, dose reduction was obliged. A rash ≤ grade 2 was managed by maintaining the current dose level, while initiating/intensifying therapy with antihistamines or topical corticosteroids. If the rash was grade 3, copanlisib was omitted until resolution to ≤ grade 1. If resolution time was ≤7 days, the dose was reduced by 1 level. The dose was permanently discontinued if resolution time was >7 days or if grade 4. 

#### 2.4.2. Efficacy

Radiologic tumour measurement using CT was performed every 8 weeks for the first 24 weeks and every 12 weeks thereafter. Patients were treated until disease progression, unacceptable toxicity or patient withdrawal. 

#### 2.4.3. Tumour Tissue Mutational Status 

DNA was extracted from archival diagnostic formalin fixed paraffin embedded (FFPE) tumour tissue using QIAamp DNA FFPE Tissue Kit (Qiagen, Hilden, Germany) after quality control by a pathologist. Mass spectrometry single nucleotide polymorphism genotyping technology (Agena Biosciences, San Diego, CA, USA) was applied to the DNA to detect a total of 37 nonsynonymous somatic mutations in *PIK3CA*. Hotspot mutations in exon 1 (R88Q, K111N), exon 4 (N345K), exon 7 (C420R, E453K), exon 9 (E542V/G/K/Q, E545K/Q/D/A/G/V, Q546H/L/P/R/E/K) and exon 20 (Y1021H/N/C, R1023Q, T1025I/A/S, A1035V/T, M1043V/I, A1046V, H1047R/L/Y, G1049R) of *PIK3CA* were analysed. 

#### 2.4.4. Circulating Tumour PIK3CA Mutation Status 

Blood samples were collected at screening and on days 1 and 15 of all treatment cycles. Plasma was isolated and DNA was extracted using a plasma/serum circulating DNA purification mini kit (Norgen Biotek, Thorold, ON, Canada). The presence and quantification of *PIK3CA* mutation hotspots E545K, E542K and H1047R was assessed with digital droplet PCR (ddPCR, Bio-Rad Laboratories, Hercules, CA, USA) using the QX200 ddPCR system (Bio-Rad Laboratories, Hercules, USA), according to the manufacturer’s instructions. QuantaSoft version 1.6.0 software (Bio-Rad Laboratories) was used for data analysis. Each plasma sample was analysed in duplicate, and the results were based on mean DNA concentrations.

#### 2.4.5. Pre and Post Treatment Biopsies

Voluntary pre and end of study tumour biopsies with matched whole blood sample were collected and snap frozen. A pathologist reviewed samples to ensure adequate tumour content. DNA was extracted using an AllPrep DNA mini kit (Qiagen, Hilden, Germany), and from whole blood using a DNA blood mini kit (Qiagen). Exome capture was performed on 500 ng DNA using the Agilent SureSelect Human All Exome V5 kit, according to the manufacturers protocol (Agilent, Santa Clara, CA, USA). Samples were sequenced to a minimum of 90X coverage (range 57–124) using 91-bp paired end reads on the Illumina HiSeqX, at the Beijing Genomics Institute (Tai Po, Hong Kong). FASTQ files were processed with Trimmomatic [20] and were aligned to the hg38 reference genome using bwa mem with default settings [21]. Duplicate reads were marked using Picard (https://broadinstitute.github.io/picard/) (accessed on 27 June 2019)) and base quality scores were recalibrated using GATK version 4 [22]. Somatic SNVs (single-nucleotide variants) and Indels were called using samtools mpileup [23] and VarScan 2 [24] and annotated with Varient Effect Predictor [25]. Copy number alterations were identified using FACETS (Fraction and Allele-Specific Copy Number Estimates from Tumor Sequencing) [26]. 

## 3. Results

Between August 2016 and July 2017, 12 patients were enrolled (Table 1). All patients who received a dose of copanlisib plus trastuzumab were assessed for safety. Patients were representative of a heavily pre-treated population with a median number of three prior therapies in the metastatic setting (range, 1–9). More than half of the participants had experienced disease progression on both pertuzumab and trastuzumab emtansine (TDM1) as well as trastuzumab prior to entering the trial. 

### 3.1. Safety

There were no DLTs observed during the first cycle of treatment at either dose level of copanlisib (45 mg (n = 6) or 60 mg (n = 6). Therefore, in combination with trastuzumab, the MTD of copanlisib was defined as copanlisib 60 mg on Days 1, 8 and 15 of a 28-day cycle. No patients required a dose reduction to dose level-1. There were 10 (7%) copanlisib dose interruptions due to infusion related hypertension (n = 6), dry skin (n = 1), fatigue (n = 1), lung infection (n = 1) and rash maculo-papular (n = 1). There were no study therapy discontinuations due to adverse events. No dose modifications were required. 

There were 11 reported serious adverse events (SAE) occurring in 7 (58%) participants. Of these, five (71%) had treatment with 45 mg copanlisib and accounted for eight of the reported SAEs; abdominal pain and lung infection were considered possibly related to trial therapy. The remaining six events at dose level 1 and the three SAEs reported in the copanlisib 60 mg (dose level 2) cohort were considered unrelated to therapy. Details of SAEs are shown in Table S2. 

Adverse events (AEs) of grade 3 or higher were relatively uncommon in both dose levels (Table 2). Two of the most common adverse events were hyperglycaemia and hypertension reflecting an on-target effect of copanlisib. Although frequent, most events occurring for hyperglycaemia were less than grade 2 and self-limiting. Patients followed a low glycemic index diet on trial. Hypertension related to infusion represented the most frequent high-grade event (grade 3: n = 4; 33%). These episodes were responsive to antihypertensive medication measures outlined in the protocol. There were no deaths during the treatment phase of the study or in the 30-day follow up after end of treatment. 

### 3.2. Anti-Tumour Activity 

**Figure 1:**
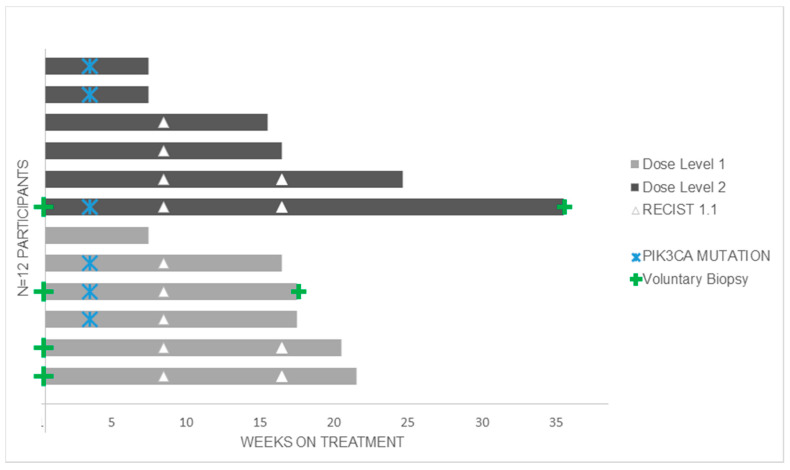
Time in weeks on copanlisib and trastuzumab.

All 12 participants were evaluable for response. The best response achieved was stable disease in nine (75%) participants at eight weeks and confirmed stable disease at 16 weeks in six (50%) of these, one of whom had a *PIK3CA* E542 mutation in archival tumour. All patients discontinued study treatment due to clinical and/or radiological disease progression. The median duration of treatment for both dose levels was 16.5 weeks (range: 7–35 weeks). The median time on treatment was 17 weeks and 15.5 weeks for dose levels 1 and 2, respectively. The median duration of stable disease was 17 weeks (range: 15–36 weeks) (Figure 1). The median follow-up time from disease progression to end of study or data cut-off was 47.9 weeks (range: 2.7–98.1 weeks). One participant withdrew from follow up after completing study treatment. 

### 3.3. PIK3CA Mutational Status in Tumour and Circulating Tumour DNA (ctDNA)

**Figure 2:**
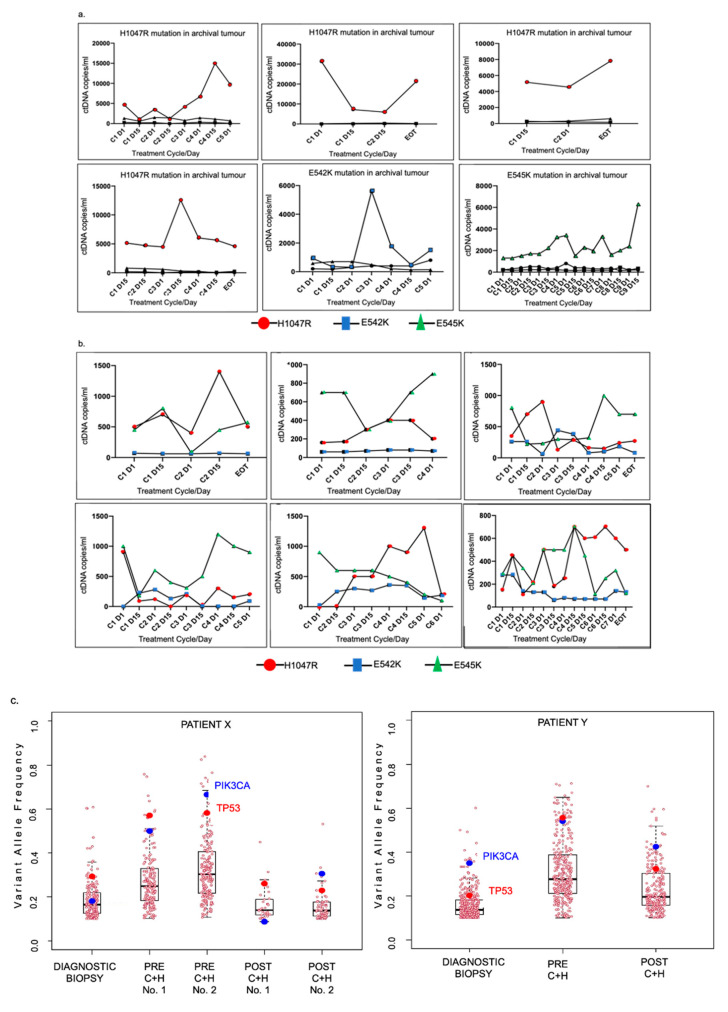
(**a**) Serial circulating PIK3CA mutant alleles (ctDNA) in patients with PIK3CA mutation in archival tumour (n = 6); (**b**) Serial Circulating PIK3CA mutant alleles (ctDNA) in patients with no PIK3CA mutation in archival tumour (n = 6); (**c**) changes in the variant allele frequency in serial tumour samples.

Six (50%) of 12 patients were found to have one of three *PIK3CA* hotspot mutations in their tumour sample (Figure 1 + Table S3), however there was no difference in response to treatment between patients with PIK3CA wildtype (WT) and PIK3CA mutated tumours. All participants, regardless of tumour *PIK3CA* mutation status, had detectable *PIK3CA* mutations in their plasma ctDNA at varying quantities. The six patients with a *PIK3CA* mutation detected in their archival tumour had the corresponding *PIK3CA* mutation detected in their pre-treatment plasma ctDNA (at 920–31,300 copies/mL plasma) and in all on-treatment ctDNA samples (Table S1). Notably, half (n = 3) of these also had >500 copies/mL of a second *PIK3CA* mutation detectable in their baseline ctDNA sample, although in lesser quantities than the tumour mutation (Figure 2). Interestingly, all six patients with *PIK3CA*-wildtype archival tumour had detectable circulating *PIK3CA* mutations in their baseline plasma ctDNA. In all participants, the number of copies of *PIK3CA* mutations in ctDNA fluctuated over the course of treatment, with no clear trend in relation to treatment response or duration. A higher peak quantity of mutant *PIK3CA* alleles in ctDNA did not necessarily appear to correlate with a shorter survival (Figure 2). 

### 3.4. Serial Tumour Biopsy Sequencing

Two participants had voluntary tumour biopsies pre- and post-clinical trial with sufficient tumour material for whole exome sequencing (WES) along with archival tumour from initial diagnosis.

In both patients, fewer than 20% of the somatic gene mutations detected, including predicted functional (deleterious) and non-functional mutations, were common to all three timepoints (Figure S1). Most mutations were unique to one or two but not all three timepoints in both patients reflecting significant temporal genomic heterogeneity. 

In one patient (patient X), two tumour biopsies were obtained at each of the pre and post copanlisib plus trastuzumab time points. In the pre-trial biopsies, 80/98 (81.6%) somatic mutations were shared while only 10/33 (30.3%) of somatic gene mutations were common to the two tumour biopsies taken post-trial, possibly reflecting more intra-tumoural heterogeneity as the tumour evolves. 

A number of secondary somatic gene mutations that are predicted to be deleterious emerged specifically post treatment with copanlisib (i.e., were only present in the post clinical trial tumour biopsies). Several of these genes have functions related to protein kinase signalling or transcriptional regulation, including *AATK, ADCY8, BMP2, CDH19, GFI1, MAVS, PDE4C, SP4* and *WAS* (Figure S1).

Interestingly, four mutated genes were common to the tumours of both patients X and Y. These were the known cancer genes *TP53* and *PIK3CA*, as well as the novel genes *DNAH3* and *TRRAP,* with the latter encoding a PI3K-like protein kinase [27]. *TP53* and *PIK3CA* were mutated in the diagnostic biopsy, pre-copanlisib biopsy and the post-copanlisib biopsy in both patients X and Y. *DNAH* was mutated in at least one biopsy sample from each patient. *TRRAP* was mutated in the pre- and post-copanlisib biopsies in patient Y and the pre-copanlisib biopsy in patient X.

There was a significant decrease in the variant allele frequency (VAF) of mutant *PIK3CA* in Patient X from pre to post-trial, suggesting possibly that copanlisib may have inhibited the tumour clone bearing the *PIK3CA* mutation and also highlighting potential heterogeneity of response between the tumors from patient X and Y (Figure 2).

## 4. Discussion and Conclusions

This first-in-human trial of copanlisib and trastuzumab demonstrates that the combination can be administered safely in patients with advanced HER2-positive, trastuzumab-resistant breast cancer. No dose limiting toxicities emerged and no unexpected novel toxicities related to the combination were reported. 

Hyperglycaemia was a common and expected adverse event related to copanlisib infusion, reflecting the and on target effect of PI3K inhibition related to insulin signal dysregulation. Prior trials involving copanlisib have reported grade 3 hyperglycaemia in up to 49% of patients [19,28]. Hyperglycaemia events on the day of infusion in this trial were primarily grade 2 and patients followed a low glycemic index diet on the day of infusion and for 48 h afterwards, which may have supported this [29]. 

In *PIK3CA* mutation positive tumours, the tissue mutation was predominant in plasma ctDNA. However, the presence of *PIK3CA* mutations in plasma ctDNA of all participants may reflect some temporal heterogeneity and suggests that *PIK3CA* mutation frequency in HER2-positive breast cancer may be underestimated by archival tissue testing. The detected quantity of mutant alleles fluctuated over the course of treatment with copanlisib and trastuzumab, suggesting a possible on-target effect of PI3K inhibition that may have acted as an indicator of disease suppression, however there was no clear pattern to the variability in quantity of detected mutant alleles over the course of treatment. The potential utility of *PIK3CA* mutations in ctDNA as biomarkers will require further study in phase 2 and 3 trials in this setting. 

We identified novel somatic mutations in the *TRRAP* gene, encoding a PI3K-like protein kinase, in tumour samples obtained at metastatic timepoints from both patients in whom NGS was performed, suggesting that *TRRAP* mutations may play a role in the development of metastatic disease. A previous study in breast cancer has indicated the TRRAP may act as a tumour suppressor and may be a potential therapeutic target [30]. Additionally, WES of sequential tumour biopsies demonstrated clear temporal genomic heterogeneity. Finally, the *PIK3CA* VAF differed between pre- and post-copanlisib tumour, suggesting a possible inhibitory effect of copanlisib on the *PIK3CA*-mutated clone.

Overall, we determined a recommended phase 2 dose for this novel combination of copanlisib in combination with trastuzumab and this trial is now ongoing. No dose limiting toxicities emerged and no unexpected novel toxicities related to the combination were reported. Results of serial genomic analysis are provocative and worth further exploration. 

## Figures and Tables

**Table 1:** Patient characteristics at baseline.

Characteristic	Copanlisib + Trastuzumab
	N = 12
Age at Therapy Start	53 (42–72)
Histology	
Ductal	11 (92%)
Lobular	1 (8%)
Receptor Status	
HER2 Positive	12 (100%)
Oestrogen Receptor Positive	9 (75%)
Prior Neo–or Adjuvant Chemotherapy	10 (83%)
Sites of Disease	
Liver Lung Bone Other	3 (25%) 3 (25%) 6 (50%) 6 (50%)
Time from Metastatic Disease to Trial Registration	
Median (range)	31 (10–140)
Prior Lines of Chemotherapy in the Metastatic Setting	
Median (range) Trastuzumab Pertuzumab TDM1 Lapatinib	3 (1–9) 12 (100%) 10 (83%) 8 (67%) 4 (33%)

HER2—Human epidermal growth factor receptor 2; TDM1—Trastuzumab Emtansine.

**Table 2:** All grade adverse events occurring in more than one patient regardless of attributed causality *.

Adverse Event	Any Grade n = 12	Grade 3 or Higher n = 12	Any Grade Dose Level 1 n = 6	Grade 3 or Higher Dose Level 1 n = 6	Any Grade Dose Level 2 n = 6	Grade 3 or Higher Dose Level 2 n = 6
Any Adverse Event n (%)	12 (100%)	7 (58%)	6 (100%)	4 (67%)	6 (100%)	3 (50%)
Hyperglycaemia	7 (58%)	0	4 (67%)	0	3 (50%)	0
Constipation	7 (58%)	0	3 (50%)	0	4 (67%)	0
Fatigue	7 (58%)	1 (8%)	4 (67%)	1 (17%)	3 (50%)	0
Hypertension	6 (50%)	4 (33%)	5 (83%)	3 (50%)	1 (17%)	1 (17%)
Nausea	7 (58%)	0	3 (50%)	0	4 (67%)	0
Diarrhea	6 (50%)	0	3 (50%)	0	3 (50%)	0
Rash	6 (50%)	0	3 (50%)	0	3 (50%)	0
Vomiting	7 (58%)	0	4 (67%)	0	3 (50%)	0
Cough	4 (33%)	0	3 (50%)	0	1 (17%)	0
Mucositis	5 (42%)	0	4 (67%)	0	1 (17%)	0
Decreased appetite	4 (33%)	0	3 (50%)	0	1 (17%)	0
Dry skin	4 (33%)	0	3 (50%)	0	1 (17%)	0
Fever	4 (33%)	0	3 (50%)	0	1 (17%)	0
Headache	4 (33%)	0	1 (17%)	0	3 (50%)	0
Paresthesia	3 (25%)	0	1 (17%)	0	2 (33%)	0
Weight decreased	3 (25%)	0	3 (50%)	0	0	0
Anemia	2 (17%)	0	1 (17%)	0	1 (17%)	0
Dehydration	2 (17%)	0	2 (33%)	0	0	0
Dyspnea	2 (17%)	1 (8%)	1 (17%)	0	1 (17%)	1 (17%)
Oedema peripheral	1 (8%)	0	1 (17%)	0	0	0
Insomnia	2 (17%)	0	1 (17%)	0	1 (17%)	0
Paresthesia Oral	2 (17%)	0	0	0	2 (33%)	0
Peripheral neuropathy	2 (17%)	0	1 (17%)	0	1 (17%)	0
Blood Bilirubin increased	1 (8%)	1 (8%)	0	0	1 (17%)	1 (17%)
Gamma GT increased	1 (8%)	1 (8%)	0	0	1 (17%)	1 (17%)
Bile duct obstruction	1 (8%)	1 (8%)	0	0	1 (17%)	1 (17%)
Lymphangitis carcinomatosis	1 (8%)	1 (8%)	0	0	1 (17%)	1 (17%)

* If an event ≥grade 3 occurred in a single patient, the event is included here. Gamma GT–Gamma-Glutamyl Transferase.

## Data Availability

The data that support the findings of this study are available on request from the corresponding author. The data are not publicly available due to privacy or ethical restrictions.

## Supplementary Materials

The following are available online at https://www.mdpi.com/2072-6694/13/6/1225/s1, Figure S1: (a): Schematic diagram of tissue samples collected, and analysis performed. (b). (i) Comparison of somatic mutations present in three biopsies given by two participants at three different timepoints: (A) at diagnosis (B) pre-copanlisib and trastuzumab and (C) at the time of disease progression on copanlisib and trastuzumab (C + H). (ii) Venn diagram of percentage of shared somatic mutation over 3 time points in Patient X. (iii) Venn diagram of percentage of shared somatic mutation over 3 time points in Patient Y., Table S1: Inclusion and Exclusion criteria, Table S2: Serious Adverse events in patients receiving the combination of copanlisib and trastuzumab, Table S3: Plasma PIK3CA mutation status. The percentage of serial plasma samples with detectable PIK3CA mutation and the percentage of these with ≥ 500 copies/mL of mutant alleles for these hotspot mutations H1047R, E542K and E545K are shown, as analysed by droplet digital PCR (ddPCR). Plasma samples were collected at baseline and every 2 weeks while on study for all patients.

## Abbreviations

AE	Adverse Event
ANC	Absolute Neutrophil Count
CISH	Chromogenic In Situ Hybridisation
CMV	Cytomegalovirus
CT	Computed Tomography
ctDNA	Circulating Tumour DNA
CYP3A4	Cytochrome P450 3A4
ddPCR	Droplet Digital PCR
DLT	Dose Limiting Toxicity
DNA	Deoxyribonucleic Acid
ECOG	Eastern Cooperative Oncology Group
FFPE	Formalin Fixed Paraffin Embedded
FISH	Fluorescence In Situ Hybridisation
GCP	Good Clinical Practice
HER2	Human Epidermal Growth Factor Receptor 2
HIV	Human Immunodeficiency Virus
HPRA	Health Products Regulatory Authority of Ireland
IHC	Immunohistochemistry
IV	Intravenous
MTD	Maximum Tolerated Dose
NGS	Next Generation Sequencing
PI3K	Phosphoinositide-3 Kinase
RECIST	Response Evaluation Criteria in Solid Tumours
SAE	Serious Adverse Event
T-DM1	Trastuzumab Emtansine
WES	Whole Exome Sequencing
WT	Wildtype
VAF	Variant Allele Frequency
