# Supplementary material for: Phase Ib Trial of Copanlisib, A Phosphoinositide-3 Kinase (PI3K) Inhibitor, with Trastuzumab in Advanced Pre-Treated HER2-Positive Breast Cancer “PantHER”
Source: Cancers (Basel). 2021 Mar 11;13(6):1225. doi: 10.3390/cancers13061225 (PMC7999809; doi:10.3390/cancers13061225)
Supplement: Supplementary file 1 [file cancers-13-01225-s001.pdf]

cancers

MDPI

Article

Phase Ib Trial of Copanlisib, A Phosphoinositide-3 Kinase (PI3K) Inhibitor, with Trastuzumab in Advanced Pre-Treated HER2-Positive Breast Cancer "PantHER"

Niamh M. Keegan, Simon J. Furney, Janice M. Walshe, Giuseppe Gullo, M. John Kennedy, Diarmuid Smith, John Mc Caffrey, Catherine M. Kelly, Keith Egan, Jennifer Kerr, Mark Given, Peter O'Donovan, Andres Hernando, Ausra Teiserskiene, Imelda Parker, Elaine Kay, Angela Farrelly, Aoife Carr, Giulio Calzaferri, Ray McDermott, Maccon M. Keane, Liam Grogan, Oscar Breathnach, Patrick G. Morris, Sinead Toomey and Bryan T. Hennessy

Supplementary Materials:

Table S1. Inclusion and Exclusion criteria

Inclusion	Exclusion
• Adult women ≥ 18 years of age<br>• Histologically confirmed HER2-positive breast cancer<br>• Recurrent incurable or metastatic breast cancer<br>• At least one measurable lesion according to RECIST criteria (Version 1.1). Patients with bone only disease are not eligible.<br>• Patient has received at least one trastuzumab-based or T-DM1-based treatment regimen in the setting of metastatic disease or incurable locoregional recurrence. A trastuzumab based or T-DM1-based treatment regimen is considered as any treatment regimen that includes trastuzumab or T-DM1.<br>• Disease progression during or following at least 1 prior trastuzumab-based or trastuzumab emtansine (T-DM1) based treatment regimen in the setting of metastatic disease or incurable locoregional recurrence.<br>• ECOG performance status ≤ 2.<br>• Availability of fresh tissue and/or archival tumour tissue at screening.<br>• Adequate organ function including fasting glucose ≤120 mg/dL (≤ 6.0 mmol/L) if not diabetic or < 160 mg/dL (≤ 8.9 mmol/L) if diabetic<br>• Left ventricular ejection fraction (LVEF), at or above the Institutions lower limit of normal, as determined by ECHO or MUGA<br>• Patients must have recovered from clinically significant side effects associated with prior radiotherapy and chemotherapy with the exception of fatigue or neuropathy.	• Known breast cancer involvement of the brain, unless adequately controlled based on the clinical judgement of the treating physician.<br>• Congestive heart failure > New York Heart Association (NYHA) class II.<br>• Unstable angina (angina symptoms at rest), new-onset angina (begun within the last 3 months). Myocardial infarction less than 6 months before registration.<br>• Uncontrolled arterial hypertension despite optimal medical management (per investigator's opinion).<br>• Uncontrolled Type I or II diabetes mellitus. Defined as HbA1c > 8.5% as determined during screening laboratory assessments.<br>• Arterial or venous thrombotic or embolic events such as cerebrovascular accident (including transient ischemic attacks), deep vein thrombosis or pulmonary embolism within 3 months before registration.<br>• Non-healing wound, ulcer, or bone fracture.<br>• Active, clinically serious infections > CTCAE Grade 2 (CTCAE v4.0).<br>• known HIV, Hepatitis B, C or CMV positivity or uncontrolled intercurrent illnesses.<br>• Patients with CMV PCR positive.<br>• Patients with seizure disorder requiring medication<br>• Patients with evidence or history of bleeding diathesis. Any haemorrhage or bleeding event ≥ CTCAE Grade 3 within 4 weeks prior to the start of study treatment.

	• Proteinuria of Grade 3 or higher (CTCAE v4.0). Patient will be excluded if > 2+ on urinalysis (unless 24 hr collection shows 24 h urinary protein < 3.5g/24hrs).<br>• History or concurrent condition of interstitial lung disease of any severity, and/or severely impaired lung function (as judged by the investigator).<br>• Concurrent diagnosis of pheochromocytoma.<br>• Pregnant or breast-feeding patients. Women of childbearing potential must have a serum or urine pregnancy test performed a maximum of 7 days before start of treatment, and a negative result must be documented before start of treatment.<br>• Unresolved toxicity higher than CTCAE Grade 1 attributed to any prior therapy/procedure, excluding alopecia, peripheral neuropathy, and bone marrow parameters.<br>• Known hypersensitivity to any of the test drugs, test drug classes, or excipients in the formulation<br>• Substance abuse, medical, psychological or social conditions that may interfere with the patient’s participation in the study or evaluation of the study results.<br>• Any illness or medical conditions that are unstable or could jeopardize the safety of patients and their compliance in the study.<br>• Patients permanently withdrawn from study participation will not be allowed to re-enter the study.<br>• Excluded therapies included investigational drugs, immunosuppressive therapy, CYP3A4 inhibitors or inducers, anti- arrhythmic therapy apart from beta blockers and digoxin.

Table S2. Serious Adverse events in patients receiving the combination of copanlisib and trastuzumab.

**Serious Adverse Events N=11**	**Dose Level 1 Copanlisib 45mg**	**Dose Level 2 Copanlisib 60mg**
Possibly Related	Abdominal Pain (n = 1)<br>Lung Infection (n = 1)	-
Unrelated	Lung infection (n = 1)<br>Urinary tract infection (n = 1)<br>Infection (n = 1)<br>Pleural effusion (n = 1)<br>Seizure (n = 2)	Dyspnea (n = 1)<br>Lymphangitis carcinomatosis (n = 1)<br>Bile duct obstruction from tumour mass (n = 1),

Table S3. Plasma PIK3CA mutation status. The percentage of serial plasma samples with detectable PIK3CA mutation and the percentage of these with ≥ 500 copies/mL of mutant alleles for these

hotspot mutations H1047R, E542K and E545K are shown, as analysed by droplet digital PCR (ddPCR). Plasma samples were collected at baseline and every 2 weeks while on study for all patients.

**Number of plasma ctDNA samples**	**Tumour tissue PIK3CA**	PIK3CA H1047R ctDNA Plasma		PIK3CA E542K ctDNA Plasma		PIK3CA E545K ctDNA Plasma		Time on treatment (weeks)
		Mutant allele detectable	>500 copies/ml of mutant allele	Mutant allele detectable	>500 copies/ml of mutant allele	Mutant allele detectable	>500 copies/ml of mutant allele	
**7**	**E542K**	100%	14%	100%	57%	100%	43%	17
16	E545K	100%	19%	100%	0%	100%	100%	35
8	H1047R	100%	100%	87%	0%	100%	100%	16
7	H1047R	100%	100%	86%	0%	86%	43%	17
4	H1047R	100%	100%	75%	0%	100%	0%	7
3	H1047R	100%	100%	100%	0%	100%	33%	7
9	Wildtype	78%	11%	55%	0%	100%	67%	20
5	Wildtype	100%	80%	100%	0%	100%	40%	7
8	Wildtype	75%	63%	87%	0%	100%	63%	21
13	Wildtype	100%	54%	100%	0%	100%	31%	24
6	Wildtype	100%	0%	100%	0%	100%	67%	16
9	Wildtype	100%	22%	100%	0%	100%	44%	15

a.

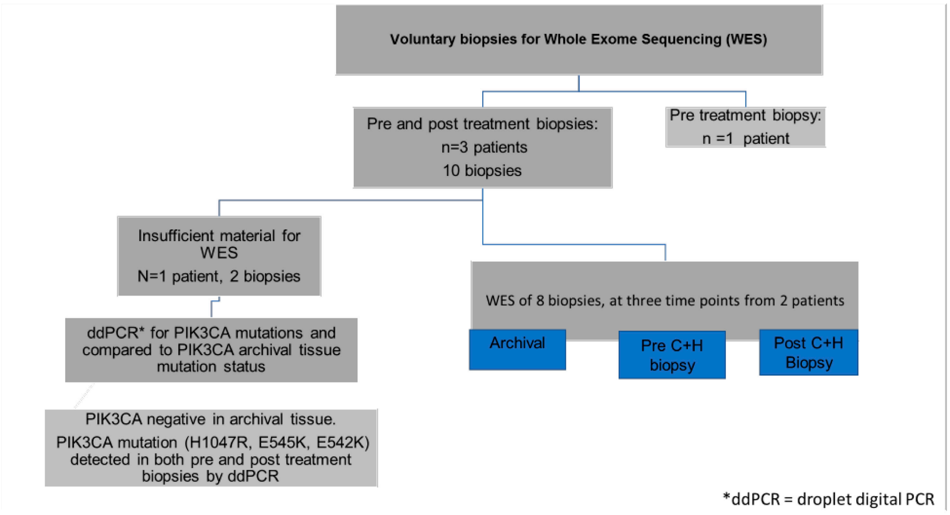

b. (i)

Number of gene mutations present (%)	Patient X	Patient Y
Total gene mutations	324	772
At all timepoints	53 (16.4%)	71 (9.2%)
At diagnosis only (A)	82 (25.3%)	355 (46%)
Pre-copanlisib only (B)	105 (32.4%)	156 (20.2%)
Post-copanlisib only (c)	17 (5.3%)	98 (12.7%)
In A and B, but not C	59 (18.2%)	13 (1.68%)
In A and C, but not B	1 (0.3%)	4 (0.5%)
In B and C, but not A	7 (2.2%)	75 (9.7%)

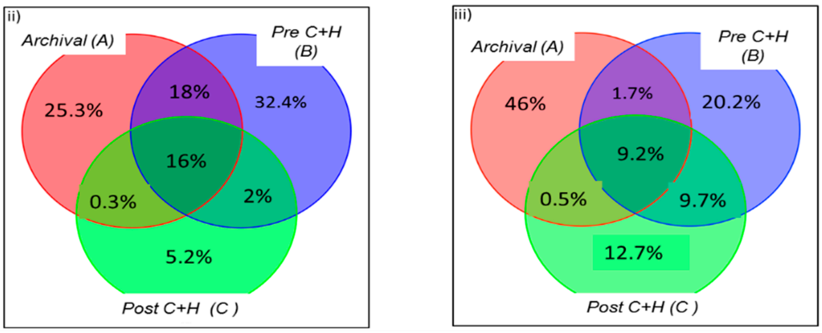

Figure S1. (**a**): Schematic diagram of tissue samples collected, and analysis performed. (**b**). (i) Comparison of somatic mutations present in three biopsies given by two participants at three different timepoints: (A) at diagnosis (B) pre-copanlisib and trastuzumab and (C) at the time of disease progression on copanlisib and trastuzumab (C + H). (ii) Venn diagram of percentage of shared somatic mutation over 3 time points in Patient X. (iii) Venn diagram of percentage of shared somatic mutation over 3 time points in Patient Y..
